# Isolation and Identification of a Novel Variant Rhabdovirus from Cultured Chinese Rice-Field Eels (*Monopterus albus*) in China

**DOI:** 10.3390/ani16071045

**Published:** 2026-03-29

**Authors:** Yan Ou, Yuzhuo He, Yiqun Li, Xin Ren, Yong Zhou, Nan Jiang, Wenzhi Liu, Yuding Fan

**Affiliations:** 1 College of Fisheries, Huazhong Agricultural University, Wuhan 430070, China; ouyan172023@163.com; 2 Yangtze River Fisheries Research Institute, Chinese Academy of Fishery Sciences, Wuhan 430223, China; swuhyzelse@email.swu.edu.cn (Y.H.); liyq@yfi.ac.cn (Y.L.); renxin@yfi.ac.cn (X.R.); zhouy@yfi.ac.cn (Y.Z.); jn851027@yfi.ac.cn (N.J.)

**Keywords:** rhabdovirus, CrERV, variant, *Monopterus albus*

## Abstract

Chinese rice-field eels (*Monopterus albus*) are a commercially farmed freshwater fish species in China. In recent years, the rapid expansion of aquaculture has been accompanied by frequent outbreaks of viral diseases, posing a serious threat to the sustainability of the Chinese rice-field eel farming industry. In this study, a rhabdovirus strain was isolated from diseased Chinese rice-field eels at a farm located in Xiantao, Hubei Province, China. Although the complete genomic sequence of CrERV-XT showed higher identity to the infectious hemorrhagic syndrome virus (IHSV) (96.16%) than to CrERV (94.39%), phylogenetic analysis based on the L protein placed CrERV-XT within the same clade as CrERV, supporting its tentative classification as a novel variant of CrERV. Furthermore, the amino acid sequence of the L protein showed greater similarity to that of CrERV (97.89%) than to IHSV, while the N, P, M and G proteins exhibited higher homology with their counterparts in IHSV. CrERV-XT displayed considerable genetic divergence from known CrERV isolates, which is presumably attributed to its geographic isolation in different locations. Alignment of the G protein sequences from five strains (CrERV-XT, CrERV, CrERV-TYY25, CrERV-XY0907 and IHSV) revealed a total of 39 amino acid mutation sites. These findings provide valuable insights for investigating conserved functional domains within the CrERV G protein for the rational design of vaccine antigens against this emerging virus.

## Simple Summary

A novel strain of the Chinese rice field eel rhabdovirus (CrERV) was found in a new city, designated as CrERV-XT. Comprehensive genomic characterization and phylogenetic analysis revealed that CrERV-XT shares 94.39% the complete genomic sequence identity with the index strain CrERV. Notably, it exhibits more genetic mutations compared to all previously characterized CrERV variants. This study enriches the pathogenic data of CrERV and provides a basis for further research into the evolution and transmission of it. Moreover, amino acid sequence alignment and antigenic epitope prediction in this study may provide useful reference and theoretical support for future research on antigen screening and prevention strategies against CrERV.

## 1. Introduction

The Chinese rice-field eel (*Monopterus albus*) is classified within the class *Actinopteri*, order *Synbranchiformes*, and family *Synbranchidae*, and in vertebrate taxonomy [1]. As a benthic freshwater fish, it is distributed across Southeast Asia, including China, North Korea, and Thailand, with a particularly extensive range in China [2]. It is an economically important freshwater aquaculture species, valued for its tender flesh, high nutritional content, and recognized medicinal properties [3]. With the advancement of the Chinese rice-field eel aquaculture, the diversity and complexity of diseases afflicting this species have progressively increased, especially viral diseases.

Earlier studies have identified several viral pathogens in *Monopterus albus*. In 2013, Ou et al. identified the *Monopterus albus* rhabdovirus (MoARV) [4]. In 2019, Liu et al. isolated and studied *Siniperhavirus chuatsi* (CrERV) from diseased fish, which has been linked to high mortality rates in affected populations [5]. More recently, in 2023, Chen et al. demonstrated that the infectious hematopoietic syndrome virus (IHSV) could jump species and infect Chinese rice-field eels, thereby adding to the known viral risks for this farmed fish [6]. All of these identified viruses belong to the *Rhabdoviridae* family and therefore share common structural and genomic characteristics. They comprise single-stranded RNA and encoding five structural proteins: the nucleoprotein (N), phosphoprotein (P), matrix protein (M), glycoprotein (G), and large protein (L) [7,8]. In the field of fish rhabdoviruses, *Novirhabdovirus piscine* (VHSV), *Novirhabdovirus salmonid* (IHNV), and *Sprivivirus cyprinus* (SVCV) have been widely researched. Because these three viruses cause major economic losses to fish farming around the world and in China, scientists have kept a close watch on them [9,10,11]. Currently, prevention and control strategies against CrERV infection mainly rely on studies of natural Chinese herbal medicines, such as arctigenin and schisandrin B (Sch B) [12,13], while no effective vaccines against CrERV have been developed to date. Identifying conserved regions and selecting appropriate antigenic epitopes by analyzing the variation among different isolates provides a valuable foundation for future studies on viral immunology and control measures. Therefore, it is imperative to conduct systematic research and analysis on distinct isolates of rhabdoviruses on Chinese rice-field eels.

In this study, pathogenic identification was conducted on diseased Chinese rice-field eels collected from Xiantao, Hubei Province, China, in April 2025. According to the on-site epidemiological investigation, the incidence rate of the farm is 30–40%, and the mortality rate within 7 days after the onset of the disease exceeds 60%. A virus was successfully isolated from the diseased tissue using the kidney of Chinese rice-field eel (CrEK) cell lines [14]. Subsequent full-genome sequencing and comparative genetic analysis confirmed that the isolated virus represents a new virus strain. This study establishes a basis for the prevention and management of viral diseases in Chinese rice-field eel and further contributes to the categorization of pathogens affecting this economically important species.

## 2. Materials and Methods

### 2.1. Cell and Fish

The kidney of Chinese rice-field eel (CrEK) cell lines [14] was established and preserved in our laboratory. It was used for all virological assays and maintained in M199 medium supplemented with 10% fetal bovine serum at 28 °C. Healthy Chinese rice-field eels (mean weight 3.5 ± 0.5 g) were obtained from an aquaculture farm in Xiantao, Hubei Province. The healthy Chinese rice-field eels had no previous disease history Fish were acclimatized for two weeks in a recirculating system at 24 ± 1 °C and confirmed free of virus and major bacterial pathogens by RT-PCR and culture prior to experimental use.

### 2.2. Sample Collection and Clinical Examination

The diseased Chinese rice-field eel (adults: 37 ± 1.0 cm, 70 ± 2.0 g) from an affected farm located in Xiantao City, Hubei Province, China exhibiting hemorrhagic skin lesions were collected. Tissue samples (liver, spleen, kidney) were aseptically collected for pathogen isolation, histopathology, and molecular analysis.

### 2.3. Bacteriological and Parasitological Examination

Liver, spleen, and kidney tissues from diseased fish were collected and streaked onto Brain Heart Infusion (BHI) agar and incubated at 28 °C for 48 h. PCR was carried out using the 16S rRNA primers, as previously described [15]. Skin and intestine scrapings were examined microscopically for parasites. Parasites in the whole-body tissues of diseased rice-field eels were screened by means of an optical microscope (DM2500; Leica, Wetzlar, Germany).

### 2.4. Cell Culture and Virus Isolation

Tissue samples harvested from diseased Chinese rice-field eels were homogenized in sterile phosphate-buffered saline (PBS) and subjected to three cycles offreezing and thawing. The homogenate was then clarified by filtration through a 0.22 μm membrane filter, and the resulting filtrate was inoculated onto CrEK cells maintained in culture at 28 °C. Cytopathological effects (CPEs) were monitored via microscopic observation, and CrEK cells exhibiting overt infection were collected to establish virus stocks. Blind passaging of the virus was conducted for three generations. Viral titers of the stocks were quantified using the 50% tissue culture infective dose (TCID_50_) assay [6].

### 2.5. Histopathological and Transmission Electron Microscopy (TEM) Observation

The liver, spleen and kidney from naturally infected eels were collected and fixed in 4% paraformaldehyde, then dehydrated and embedded in paraffin. Sections (5 μm) were rehydrated and stained with hematoxylin and eosin. Finally, sections were observed under light microscopy (DM2500, Leica, Wetzlar, Germany). Infected CrEK cells were fixed in 2.5% glutaraldehyde, post-fixed in 1% osmium tetroxide, and processed for ultrathin sectioning. Sections were stained with uranyl acetate and lead citrate and examined under a transmission electron microscopy (Hitachi-7650, Tokyo, Japan) [5].

### 2.6. Viral Genome Sequence and Analysis

Viral RNA was extracted from infected cell culture supernatant using YEASEN MolPure^®^ Viral RNA Kit (19321ES50, YEASEN, Shanghai, China). Reverse transcription was performed using random hexamers. Initial detection used primers targeting the nucleoprotein (N) gene. The complete genome was amplified via overlapping RT-PCR and rapid amplification of cDNA ends (RACE) using virus-specific primers (Table 1). PCR products were cloned into pMD19-T vector and sequenced. Open reading frames (ORFs) prediction was obtained from NCBI. Protein sequences were aligned using IBS 2.0. Phylogenetic trees based on the L protein were constructed using the neighbor-joining method in MEGA 11.0 with 1000 bootstrap replicates. Sequences were compiled and assembled using SnapGene 7.1.2.

### 2.7. Experimental Infection

Healthy eels were randomly divided into infection and control groups. Each group contained 30 individuals, and the infection experiment was performed in triplicate. The infection group received an intraperitoneal injection of 40 µL virus suspension (10^5.3^ TCID_50_/mL). The control group received sterile PBS. Eels were monitored over a 14-day in order to observe the manifestation of clinical signs and mortality. Data were plotted and analyzed for mean and standard deviation (SD) using GraphPad Prism 10. Moribund fish were sampled for virus re-isolation and RT-PCR detection.

## 3. Results

### 3.1. Clinical Manifestations and Laboratory Examinations

**Figure 1:**
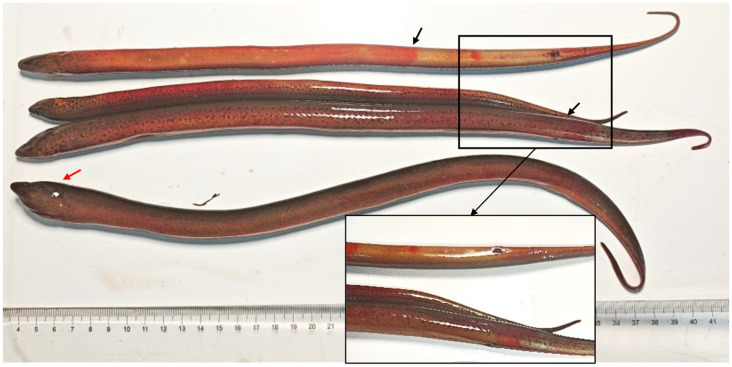
Pathological signs of Chinese rice-field eels naturally infected with the rhabdovirus. The black arrows indicate the sites of cutaneous hemorrhage and the red arrow indicate cephalic swelling.

Diseased fish presented with multiple hemorrhagic lesions on both the body surface, along with typical perianal erythema and swelling (Figure 1). Subsequent pathogen detection revealed that no pathogenic bacteria were isolated from the diseased individuals, and no parasitic infestations were identified through microscopic examination.

### 3.2. Histopathologic Observation

**Figure 2:**
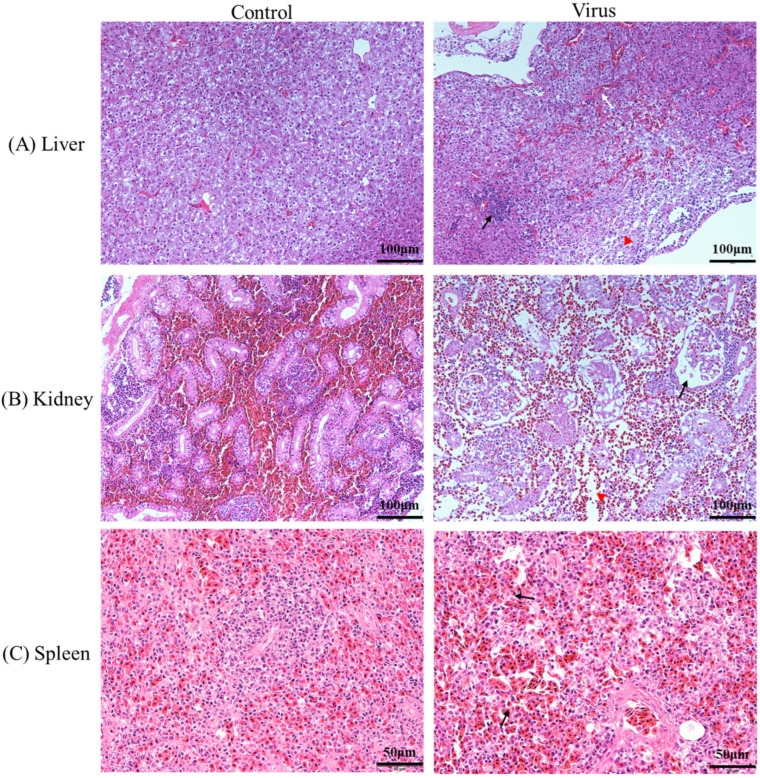
Histopathological features of different tissues from infected Chinese rice-field eels. (**A**) Liver tissue: inflammatory cell infiltration (black arrow), hepatic sinusoid swelling (white arrow) and necrotic hepatocyte (red triangle); (**B**) Kidney tissue: swell glomeruli (black arrow), renal tubules (white arrow) and the structure were indistinct or even absent (red triangle); (**C**) Spleen tissue: obvious hemorrhage (black arrow). Scale bars in (**A**,**B**), 100 μm; Scale bars in (**C**), 50 μm.

In contrast to healthy eels, histological observation of the liver, spleen and kidney tissues from diseased individuals exhibited distinct pathological alterations in the HE stained sections (Figure 2). The liver tissue showed infiltration of inflammatory cells, enlarged hepatic sinusoids, and necrotic hepatocyte (Figure 2A). The kidney tissue of the diseased fish exhibited extensive necrosis. The swollen glomeruli and renal tubules even partly disappeared (Figure 2B). The spleen tissue revealed partial necrosis as well as a significant elevation in blood cell abundance compared with the control group (Figure 2C).

### 3.3. Virus Isolation

**Figure 3:**
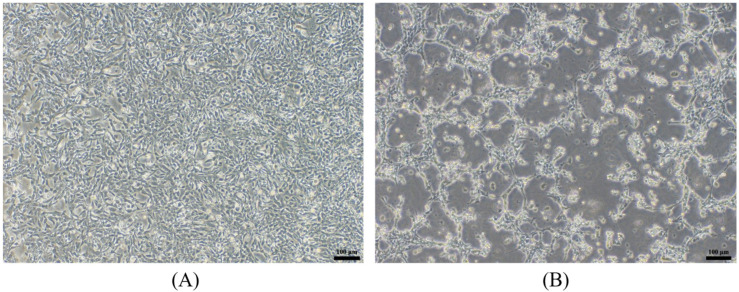
Cytopathic effect caused by the rhabdovirus (P3) in the CrEK cell line. (**A**) Control group: CrEK cells with normal morphology, no obvious CPE; (**B**) Group inoculated with the P3 virus: significant cellular pathological changes occurred, with typical CPE present.

**Figure 4:**
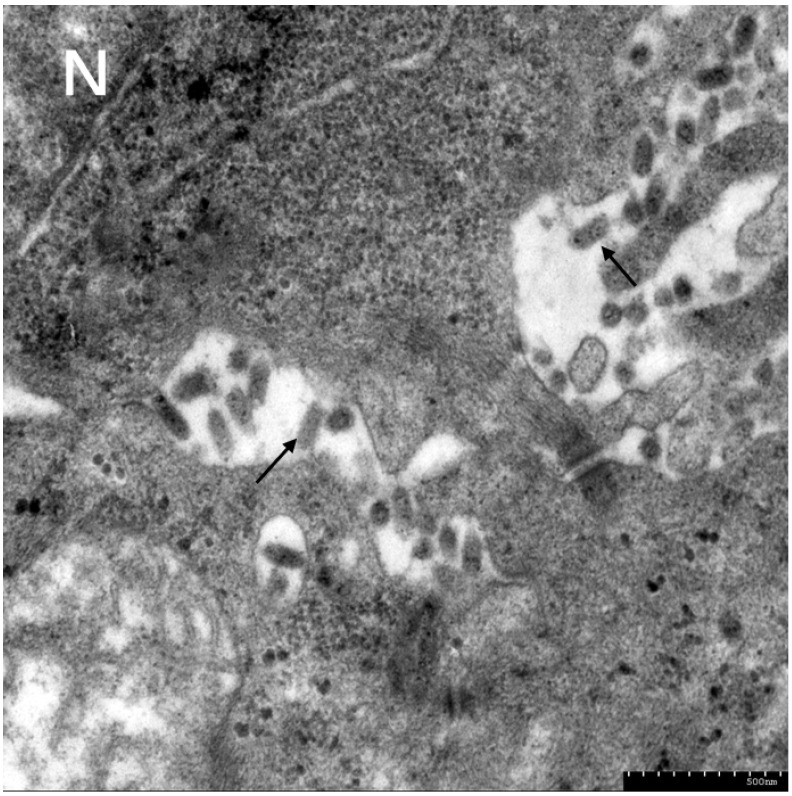
Transmission electron microscopy of the CrEK cells infected with CrERV-XT under different magnifications. Virus exhibit a typical bullet-shaped morphology in cytoplasmic vesicles (black arrows). N: nucleus; Scale bars: 500 nm.

Tissue homogenates prepared from diseased fish were subjected to filtration through a 0.22 μm filter, and the target virus was subsequently isolated via propagation on CrEK cell lines monolayers. CPE became evident within 72 h following viral inoculation (Figure 3). After three consecutive blind passages in CrEK cell lines, as shown in Figure 4, virus was concentrated in cytoplasmic vesicles and displayed a characteristic bullet-shaped morphology, about 140 nm in length and 50 nm in diameter.

### 3.4. CrERV Variant Genomic Sequence Analysis

**Figure 5:**
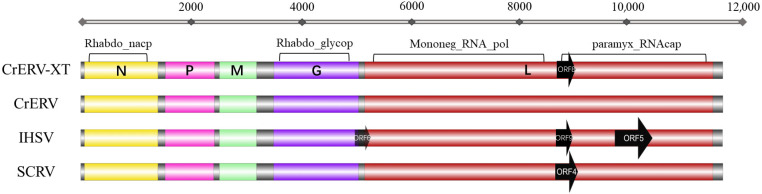
Genomic architecture and protein conserved regions of CrERV-XT. Comparison of overall genomic structures among CrERV-XT (PX990464), CrERV (MH319839.1), IHSV (OQ730265.1) and *Siniperhavirus chuatsi* (SCRV, NC_008514.1), the predicted open reading frames (ORFs) are depicted as black arrows, with the direction of transcription indicated; only predicted expressed ORFs (≥250 nt) are shown.

According to RT-PCR and RACE results, the full-length genome of CrERV-XT is 11,544 bp, and GC content is 44%. Blast analysis of the full-genome sequence against NCBI reference sequences revealed that the isolate shared 94.39% nucleotide identity with CrERV and 96.16% with IHSV. It exhibits the conserved structural hallmarks characteristic of rhabdoviruses, with genomic elements arranged in the canonical 3′-to-5′ order: nucleoprotein (N), phosphoprotein (P), matrix protein (M), glycoprotein (G), and large polymerase protein (L). This genomic architecture is conserved across members of the *Siniperhavirus* genus. Conserved domain analysis of CrERV-XT revealed that the nucleotide region spanning 139–1320 bp encodes the Rhabdovirus nucleocapsid protein (Rhabdo_ncap), a complete functional form of which is indispensable for viral encapsidation. Meanwhile, the 3518–4774 bp segment harbors the conserved domains characteristic of rhabdovirus spike glycoproteins (Rhabdo_glyco). Additionally, the conserved domains corresponding to Mononegavirales RNA-dependent RNA polymerase (Mononeg_RNA_pol) and mRNA capping enzyme (paramyx_RNAcap) are situated within the L protein, mapping to nucleotide positions 5242–8340 bp and 8632–11,283 bp, respectively (Figure 5). The Open Reading Frame Viewer from NCBI was utilized to identify ORFs. As demonstrated in Figure 5, the alignment results indicate that the number of long open reading frames of CrERV-XT on the L gene is distinct from that of CrERV and IHSV.

### 3.5. Phylogenetic Analysis and Classification

**Figure 6:**
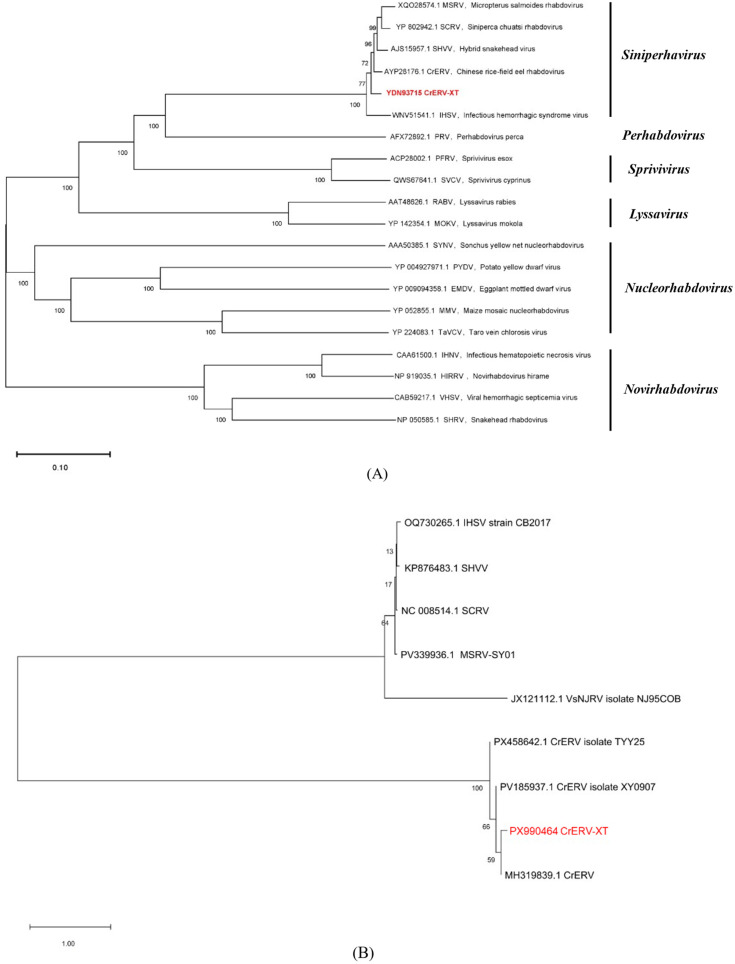
Phylogenetic analysis from different rhabdoviruses. (**A**) L protein sequence-based analysis, neighbor-joining method. (**B**) Fish rhabdovirus complete genome-based analysis, neighbor-joining method. The phylogenetic tree was constructed by using MEGA 11.0 software.

Phylogenetic analysis based on the conserved L protein sequences of rhabdoviruses (Figure 6A) revealed that the isolate CrERV-XT (GenBank accession no. PX990464) exhibited the closest evolutionary relationship with CrERV (AYP28176.1), forming a well-supported subclade. This subclade subsequently clustered with IHSV (WNV51541.1) within the genus *Siniperhavirus*, while forming distinct, independent branches from other rhabdovirus genera.

Consistent results were obtained from the phylogenetic tree constructed using complete genome sequences of representative fish rhabdoviruses (Figure 6B), where CrERV-XT consistently clustered in the same clade as CrERV, with a significantly closer evolutionary distance to CrERV than to IHSV. According to the ICTV species demarcation criteria for the genus *Siniperhavirus*, the sequence divergence between this virus and CrERV does not meet the threshold for defining a new species, and both were isolated from the same host, *Monopterus albus*. Meanwhile, CrERV-XT shared 94.39% whole-genome nucleotide identity with the reference CrERV strain (MH319839.1) and showed obvious genetic divergence from other CrERV isolates. Therefore, this virus was classified within the genus *Siniperhavirus*, tentatively identified as a novel variant of CrERV, and designated as CrERV-XT.

### 3.6. Amino Acid Sequence Alignment and Analysis

**Figure 7:**
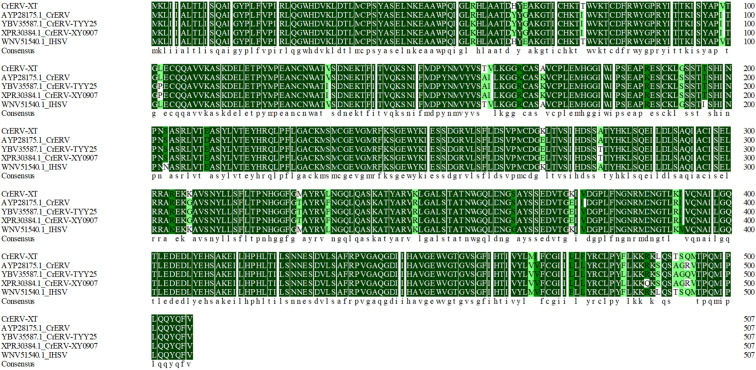
Detailed G protein sequence alignment. G protein sequence alignment of CrERV-XT with CrERV (AYP28175.1), CrERV-TYY25 (YBV35587.1), CrERV-XY0907 (XPR30384.1) and IHSV (WNV51540.1).

**Figure A1:**
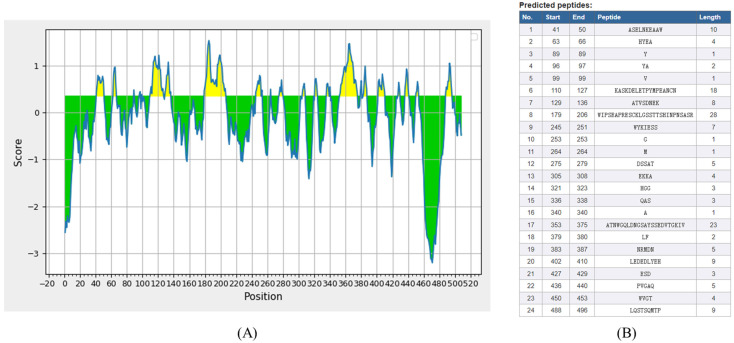
B-cell epitope prediction of CrERV-XT. (**A**) B-cell epitope prediction of the CrERV-XT G protein with IEDB. (**B**) Detailed positions, ranking and lengths of the predicted B-cell epitopes.

Sequence alignment of amino acid sequences demonstrated that the identity percentage between the N, M, P, G, L proteins of CrERV-XT and the corresponding proteins of IHSV and CrERV show in Table 2. Sequence alignment analysis revealed that the L protein of CrERV-XT exhibited the highest sequence identity with that of CrERV, whereas the amino acid sequences of its N, M, P and G proteins showed higher sequence identity with those of IHSV. A detailed analysis and alignment of the glycoprotein amino acid sequences of existing CrERV isolates and IHSV revealed that CrERV-XT harbored 39 amino acid mutation sites, with four consecutive amino acid mutations occurring at positions 491–494 aa. The amino acid sequences of the five strains were completely identical across three long fragments (1–54 aa, 210–266 aa and 392–464 aa), suggesting that the conserved fragments of the G protein are likely to be localized in these regions (Figure 7). Based on the G protein prediction results, the amino acid sequences spanning 41–50 aa, 110–136 aa, 179–206 aa, 336–387 aa and 402–453 aa show a high probability of containing B-cell epitopes (Figure A1A,B). The high predictive confidence for these segments suggests that they may serve as theoretical references and promising candidates for further experimental validation and antigen screening in future vaccine development, since they are likely to play key roles in mediating antibody recognition and host immune responses.

### 3.7. Virus Challenge

**Figure 8:**
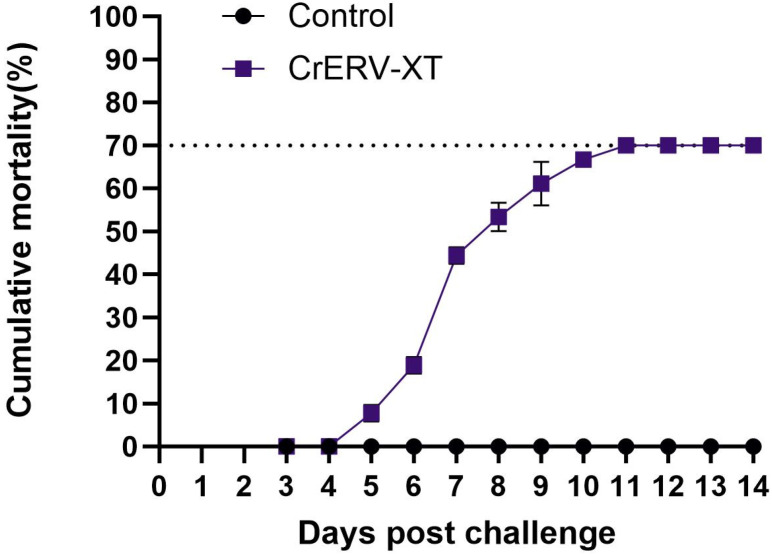
Cumulative mortality after challenge with CrERV-XT.

Mortality in the challenge group first emerged on day 4 post-challenge, peaking between days 5 to 7. As shown in Figure 8, the curve became flat after day 10, indicating that no further mortality occurred. Infected eels presented with typical hemorrhagic manifestations throughout the morbidity period. By the end of the observational period, the cumulative mortality rate of the challenge group reached 70% (Figure 8), whereas no mortalities were observed in the control group. The results of PCR detection verified the presence of rhabdovirus in dead eels from the experimental group.

## 4. Discussion

Rhabdovirus particles exhibit a characteristic bullet-shaped morphology, with dimensions of approximately 100–180 nm in length and 45–100 nm in diameter in animal hosts [16]. As a single-stranded negative-sense RNA virus, rhabdovirus is capable of inducing severe hemorrhagic symptoms in both freshwater and marine fish [17]. Additionally, interspecies transmission has been documented among fish rhabdovirus. For example, Snakehead Vesiculovirus (SHVV) is also capable of infecting *Monopterus albus* [18], while Viral Hemorrhagic Septicemia Virus (VHSV) exhibits a broad host range that encompasses multiple fish species, including salmon, trout and turbot [19]. To date, three types of rhabdovirus have been isolated using *Monopterus albus*, namely MoARV, CrERV, and IHSV [4,5,6].

In this study, a new rhabdovirus strain was isolated from CrEK cell lines and subsequently identified as the causative agent responsible for Chinese rice-field eels disease outbreak in Xiantao, Hubei Province, China, in April 2025. Histopathological examination revealed severe lesions in the liver, spleen, and kidneys of Chinese rice-field eels infected with CrERV-XT. Increased numbers of blood cells were observed in spleen sections, which was consistent with the clinical signs of organs and surface hemorrhage. The complete genome sequence of the isolate was obtained. Following whole-genome analysis, although the full-genome nucleotide sequence identity between this novel virus and IHSV (96.16%) was higher than that between this novel virus and CrERV (94.39%), phylogenetic trees constructed based on both the complete genome and the L protein sequence consistently showed that this virus is more closely related to CrERV. In accordance with the species demarcation criteria for the Rhabdoviridae family and related genera stipulated by the International Committee on Taxonomy of Viruses (ICTV), this isolate was tentatively characterized as a CrERV variant and designated CrERV-XT. CrERV-XT possesses a conserved 3′-N-P-M-G-L-5′ genomic architecture typical of other rhabdoviruses.

Fish rhabdoviruses feature small genomic size, short generation time and rapid mutation. Different variations in genes can lead to various changes in a strain. This study found that the L protein of CrERV-XT shared amino acid sequence similarities of 96.21% and 97.89% with the homologous proteins of IHSV and CrERV, respectively. This isolate was observed that the virus in question shows high similarity to viruses of the same species, and displays comparable similarity rates with those of different species. As reported by Walker [20], the L protein exhibits one of the highest conservations among all rhabdoviruses proteins. Thus, the L protein was employed in this study to construct a phylogenetic tree for evolutionary analysis. Additionally, the N protein combines with the phosphoprotein (P) and large protein (L) to form an RNA-dependent RNA polymerase complex, which is responsible for the production of viral genomic RNA and viral protein mRNAs. Based on its biological function, the preservation of this high conservation is proposed to indicate the underlying replication mechanism and basic functional framework of the virus [21,22,23]. Interestingly, apart from the L protein, the remaining structural proteins (N, M, P, G) of CrERV-XT exhibited higher identity with IHSV.

For the nucleoprotein (N), this new variant shared 98.49% sequence similarity with IHSV and 95.02% with CrERV. The N protein serves as the core structural protein of rhabdoviruses and viral nucleic acids are enclosed by it within viral particles [21]. Therefore, it is closely associated with viral RNA replication. The findings of this study indicated notable variations in the sequence conservation levels of the N protein across different viral species. Previous studies have noted that the N gene is commonly used as a marker for intra-genus typing and viral evolutionary analysis, with its genetic variations potentially correlating with viral geographic distribution [24,25]. The strain found in this study was collected from a different city than earlier known strains, which we think may be one reason for the observed gene changes. Studies have reported that the P protein works as a bridge between the L protein and the N-RNA template, keeping their correct position relationship [26]. The matrix protein (M) is located on the inner membrane surface, acting as a connector between the internal and external parts of the viral particle and playing an important role in rhabdovirus morphogenesis [23,27]. Both of these proteins in CrERV-XT share over 90% similarity with CrERV and IHSV, thus keeping the correct assembly and basic shape of rhabdoviruses.

As a key factor of host-receptor binding and viral immune evasion, the evolutionary divergence and sequence diversity of the G protein merit further in-depth investigation. Early research has involved the design of vaccine antigens via G protein analysis to identify neutralizing antigenic epitopes and glycosylation sites [28,29]. Furthermore, reverse genetics approaches have been employed to generate recombinant viruses, and the G gene has been identified as a potential virulence factor for these viruses [30]. Variations in key epitope sites may lead to neutralization escape. Comparison of G proteins across different isolates, and even among viruses of the same genus, can help evaluate the efficacy of existing vaccines and antibodies [31]. In this study, the identity of G protein between CrERV-XT and CrERV was 94.69%, which was significantly different from that of other isolates. This difference is of great importance for identifying conserved antigenic sites in the G protein and provides an essential reference sequence [31]. Combining the G protein sequence alignment results of different strains with the B-cell epitope prediction outcomes, although the regions spanning 179–206 aa and 353–375 aa displayed relatively high prediction scores, the frequent amino acid mutations at these sites may result in variations in the immunogenicity of antigenic epitopes among different strains. Based on the analysis of conserved regions and predicted antigenic epitopes of the G protein, the conserved epitopes with potential applicability for multi-strain targeting are likely localized in the regions of 41–50 aa, 110–127 aa and 379–453 aa. To date, no effective control strategies have been created for eel-infecting rhabdovirus strains such as IHSV, CrERV and MoARV. Thus, it is imperative to identify highly conserved regions and analyze potential antigenic epitopes by comparing G proteins from different isolates and viral variants, which will improve our understanding of viral antigenic variation and evolutionary characteristics. CrERV-XT supplements the dataset of distinct CrERV isolates, and the approach of identifying conserved regions shared by different CrERV strains combined with B-cell epitope analysis offers a reference for screening suitable antigens to address vaccine ineffectiveness against viral variants.

Overall, the new variant CrERV-XT has been detected alongside the wild-type CrERV in multiple cities. Its isolation and characterization have partially filled critical knowledge gaps regarding the diversity and evolutionary relationships among distinct CrERV lineages. However, a comprehensive understanding of the evolutionary dynamics, genetic diversity, and geographic distribution patterns of CrERV variants—and the correlations among these features—will require the isolation and analysis of additional strains [32,33]. The isolation and identification of viral pathogens provide a fundamental scientific basis for developing targeted prevention and control strategies against viral diseases. This work underpins the development of rapid diagnostic techniques and the formulation of precise biosecurity measures for blocking viral transmission.

## 5. Conclusions

In this study, a novel variant of CrERV, designated CrERV-XT, was first isolated from Xiantao. The whole genome of CrERV-XT shares 94.39% homology with the prototype strain CrERV. There are 39 amino acid mutation sites in the G protein, among which 4 are consecutive mutations. Phylogenetic analysis and L protein comparison confirmed that it is a variant of CrERV. B-cell epitope prediction suggested that multiple conserved regions could be used as candidate targets for vaccine design. Collectively, these findings advance our understanding of the genetic diversity of rhabdovirus infecting Chinese rice-field eels and provide a scientific basis for the screening of conserved and effective antigens and vaccine design.

## Figures and Tables

**Table 1:** Virus-specific primers used in this study. CrERV-race for amplifying the 3′ and 5′ terminal, CrERV-1 to CrERV-9 for the genome of CrERV-XT.

Name	Forward Primer	Reverse Primer	Genome Position (bp)
CrERV-race-5′	CTAATACGACTCACTATAGGGCAAGCAGTGGTATCAACGCAGAGT	CAAGGCCCTTGTGATCCTGTTGTTGGG	1–501
CrERV-race-5′	CTAATACGACTCACTATAGGGC	CCCTTACTCTGCCACAAAGAACCCAGGA	1–993
CrERV-race-3′	CCCGATGGCCACAGGAGCACATTA	CTAATACGACTCACTATAGGG-CAAGCAGTGGTATCAAC-GCAGAGT	10,043–11,544
CrERV-race-3′	CCCGGAACCAGTCCTCCTTCCATCAG	CTAATACGACTCACTATAGGGC	10,955–11,544
CrERV-1	GACATTGTGGTCCGCTATCTCTA	CCATTCTCCTCAGTCCTTCCTTC	310–1324
CrERV-2	GCTGAAGTGGAGAGGATGATGAAA	AGGTGCTTGATACGGCTTAATAGC	871–2591
CrERV-3	GAAAACTCCCAGCCGAAAATTGA	CTGTCGCAGCGAGATGTCTCAAT	2332–3656
CrERV-4	CCGACTCCATTAATGCAACATTCAC	CATGTATGATATCGCCTTGGGC	3274–4808
CrERV-5	TTCAATGGGAATCGGATGGACAAT	GTGTGTTCAGAGACATGCTGGTAG	4610–6425
CrERV-6	ACCTATCACAAGAGCACCCAGAAG	TGTGTTGTTGTAGACCACCTTCC	6115–7884
CrERV-7	TCAGTCTCAACAAATGCTCTAACC	CTCTTGATTCCAGGACTACCTCTTC	7576–9052
CrERV-8	ACCTTTAGGGATATAGGGACCGA	TTTGGATTTCTTCGTTTCCCAGTGC	8875–10,628
CrERV-9	CTTCAGAAACAGTCAAGCAGCGG	CATGGGACGAGAAAAACAAACAC	9965–11,544

**Table 2:** Amino acid sequence alignment results of each protein of CrERV-XT with CrERV and IHSV. Analyzed by Geneious Prime 2025.1.

	Virus	CrERV	IHSV
Protein			
N		95.02%	98.49%
P		93.73%	98.96%
M		94.71%	99.04%
G		94.69%	98.43%
L		97.89%	96.21%

## Data Availability

The original contributions presented in this study are included in the article. Further inquiries can be directed to the corresponding authors. Requests for materials should be addressed to Yuding Fan.
